# Comparing Neural Networks and Naive Bayes in the Prediction of Drug Gene Interactions of Type 4 Collagenase for Gingival Epithelialization

**DOI:** 10.1155/ijod/7501904

**Published:** 2026-02-20

**Authors:** Shreya Arya, Deepavalli Arumuganainar, Pradeep Kumar Yadalam, Carlos M. Ardila

**Affiliations:** ^1^ Saveetha Medical College and Hospitals, Saveetha Institute of Medical and Technical Sciences, Saveetha University, Chennai, India, saveetha.com; ^2^ Department of Periodontics, Saveetha Dental College and Hospitals, Saveetha Institute of Medical and Technical Sciences, Saveetha University, Chennai, India, saveetha.com; ^3^ Faculty of Dentistry, Biomedical Stomatology Research Group, Universidad de Antioquia U de A, Medellín, Colombia, udea.edu.co

**Keywords:** drug–gene interactions, gingiva, machine learning, neural networks, type 4 collagen

## Abstract

**Objective:**

This study aims to compare the predictive abilities of neural networks and Naive Bayes in forecasting drug–gene interactions of type 4 collagenase in gingival epithelialization.

**Materials and Methods:**

This study examines drug–gene interactions in type 4 collagen using a dataset encompassing drugs, genes, biochemical activity, mode of action, and molecular activity. Data normalization and handling of missing values are conducted to minimize the influence of larger variables. Machine learning algorithms such as neural networks and Naïve Bayes forecast or categorize drug–gene interactions. The neural network architecture, featuring 10 hidden layers, the ReLU activation function, the Adam optimizer, regularization, and a maximum of 100 iterations, is adept at solving complex problems.

**Results:**

Naive Bayes demonstrates a high area under the curve of 0.995 and notable classification accuracy (CA), but registers low overall accuracy and F1 score. It outperforms the Neural Network model in accuracy, precision, F1 score, and recall, but exhibits low specificity, potentially leading to elevated false‐positive rates.

**Conclusion:**

Predictions of drug–gene interactions for type 4 collagen hold promise for understanding biological pathways, identifying drug targets, designing targeted therapies, understanding disease mechanisms, and facilitating personalized medicine. The predictive models employed provide potential applications in personalized medicine, facilitating targeted therapies and disease management strategies. By elucidating biological pathways and drug targets, this research holds promise for advancing clinical interventions and improving patient outcomes in oral health care.

## 1. Introduction

The gingival epithelium, specialized epithelial tissue in the oral cavity, is vital for maintaining the integrity and function of the extracellular matrix (ECM) [1]. Type 4 collagenase, an enzyme, is crucial for tissue remodeling, inflammation regulation, and overall oral health by breaking down damaged collagen, a major basement membrane component providing structural support to the underlying connective tissue [1, 2]. Fibroblasts and inflammatory cells secrete type 4 collagenase, facilitating the breakdown of damaged collagen in the ECM, regulating the inflammatory response, and triggering immune responses in the gingival epithelium [3]. Dysregulation of this enzyme can lead to pathological conditions such as periodontitis. Factors influencing its expression include bacterial toxins, inflammatory mediators, anti‐inflammatory molecules, and certain medications, and understanding these mechanisms could aid in identifying therapeutic interventions for oral health [4, 5].

Type IV collagen and laminin are essential components of the basement membrane, crucial for maintaining the structural integrity of the epithelium [2]. The α6 chain of Type IV collagen regulates the expression of keratin 10, a protein crucial for oral mucosal epithelium keratinization [2, 4, 6]. Previous studies in mice have shown that α5(IV) and α6(IV) chains are present in keratinized mucosa, with α5 (IV) and α6 (IV) knockout mice exhibiting delayed epithelial development and reduced KRT10 levels [6, 7]. Keratinized mucosa is vital for maintaining healthy gingival tissue, and understanding oral mucosa keratinization is crucial for managing healthy gingiva.

Type IV collagen, a network‐forming collagen present in tissue basement membranes, plays a crucial role in preserving the integrity and health of periodontal tissue. It comprises six genetically distinct chains that form a mesh‐like structure supporting cell adhesion, migration, and differentiation [1, 6]. The integrity of Type IV collagen is indispensable for maintaining periodontal health, as it contributes to the mechanical stability of gingival tissue and regulates the exchange of molecules and cells between the epithelium and connective tissue.

Type IV collagen is a vital component of the gingival epithelium, providing structural support, facilitating cell adhesion, and maintaining tissue integrity. It also contributes to the epithelial barrier’s protection and homeostasis. It may regulate cell proliferation, differentiation, and survival, influencing immune responses and tissue regeneration and aiding in wound healing in injury or inflammation.

Various types of periodontal diseases, including gingivitis and periodontitis, are linked to alterations in the composition or structure of Type IV collagen. Enzymatic degradation of this collagen, mainly by matrix metalloproteinases, markedly increases in periodontal disease [8, 9]. Understanding the role of Type IV collagen in the gingival epithelium has significant therapeutic implications, including developing strategies to preserve or restore its integrity and exploring the potential of collagen‐based scaffolds for tissue regeneration. Type IV collagen drugs include collagenase‐based therapies, antifibrotic agents such as pirfenidone and nintedanib, gene therapy using viral vectors, monoclonal antibodies targeting specific proteins, and matrix‐based therapies for tissue regeneration or repair. The challenges in drug development include targeting type IV collagen, regulatory approval requirements, individual variation, and cost and accessibility.

Drug–gene interactions involve genetic variations that impact an individual’s response to a drug, affecting drug metabolism, transporters, and targets, thereby influencing drug efficacy, toxicity, and sensitivity. One previous study proposes a pharmacokinetic‐DDIP model that accurately predicts fold changes in area under the time–concentration curve, using FDA drug labels. The model predicts fold changes within ±0.5959, with good performance confirmed by external validation. The study introduces a new drug interaction prediction method using a simplified drug target profile representation and an L2‐regularized logistic regression model. It uses statistical metrics to evaluate drug interactions, revealing that drugs are more likely to interact when they share target genes, have short pathways, or are part of cross‐talk signaling pathways, providing insights into potential adverse drug reactions.

Previous studies showed that C4M, a marker of type IV collagen metabolism, was measured in patients with rheumatoid arthritis in two phase III studies. Type IV collagen remodeling was associated with disease activity and radiographic progression in RA and was persistently and dose‐dependently suppressed by tocilizumab. Understanding these interactions enables healthcare professionals to identify patients who require dosage adjustments or alternative treatments. It informs the development of new drugs tailored to specific genetic profiles, enhancing effectiveness and minimizing adverse effects [10–12].

Prediction of drug–gene interactions, protein–protein interactions (PPIs), single protein function, and protein family classification is crucial in biomedical research and drug discovery. These predictions aid in identifying potential drug targets, understanding drug mechanisms, and optimizing treatment strategies. Single protein function is essential for identifying enzymes, receptors, and transporters. In contrast, protein family classification categorizes proteins into structurally and functionally related groups, providing functional annotations and identifying new protein families with potential therapeutic implications [13–15].

These advancements in predictive techniques promote personalized medicine, facilitate the design of targeted therapies, and deepen our understanding of molecular mechanisms across diverse biological contexts. However, the accuracy of predictions can vary, and more comprehensive genomic data and larger datasets with clinical outcomes are needed to enable more precise predictions.

This study uses neural networks and Naive Bayes models to predict drug–gene interactions related to Type IV collagenase in gingival epithelialization. It addresses a gap in existing research by exploring the unique biochemical context of this enzyme. The approach aims to establish a predictive framework for personalized therapeutic strategies in oral health, enhancing understanding of periodontal disease and tissue regeneration.

The study introduces a new method for predicting drug–gene interactions related to Type IV collagenase in gingival epithelialization, a topic underexplored in the literature. It uses a dual‐method approach to evaluate predictive performance and establish a framework for personalized oral health treatments, focusing on individual genetic profiles. The study fills a gap in predicting drug–gene interactions in Type IV collagen, enhancing our understanding of complex biological pathways and potentially improving treatment outcomes. It contributes to a broader understanding of drug–gene interactions, which is crucial for developing safer therapies.

There are limited studies on predicting drug–gene interactions, specifically for type 4 collagen drugs. This study is an exploratory computational investigation assessing the feasibility and behavior of predictive models for drug–gene interactions related to Type IV collagenase–mediated gingival epithelialization. Neural networks learn patterns within data, whereas Naive Bayes employs probabilistic models to learn and predict outcomes for biological datasets. Therefore, our study aims to compare the predictive performance of neural networks and Naive Bayes in forecasting drug–gene interactions of type 4 collagenase in gingival epithelialization.

## 2. Materials and Methods

### 2.1. Preparation of the Dataset

Drug–gene interactions involve retrieving a type 4 collagen signaling drug–gene interaction dataset from the Probes & Drugs database, annotating and pre‐processing it, and constructing a drug–gene interaction graph using a network analysis tool such as Cytoscape [16, 17]. This dataset comprises information on drugs, genes, biochemical activities, modes of action, and molecular activities for analysis. Drug–gene interactions involving type IV collagen were gathered from https://www.probes-drugs.org. The dataset includes 205 pharmacological compounds targeting specific genes and their proteins. Before analysis, drug–gene interactions were preprocessed and outliers removed. It provides info on mechanisms of action, PPIs, modes of action, and protein complex groups related to a protein family. Sourced from the Probes & Drugs database, it focuses on Type IV collagen–related genes. Feature engineering involved annotating biochemical activity, mode of action, and molecular activity, with normalization to reduce bias. Missing data was excluded, and the dataset was split 80/20 into training and testing sets for unbiased evaluation.The biological context focused on enzymatic regulation, ECM remodeling, and epithelial integrity relevant to the gingival tissue. Before model training, feature distributions were examined to assess class imbalance and variability, confirming a skewed distribution typical of biological interaction datasets, thereby justifying the use of complementary performance metrics beyond accuracy alone.

### 2.2. Related Work

**Table:** 

Method	Features	Performance metric	Key finding
Attention networks and others	Drug structure, protein sequence, known interactions	AUROC: 0.978	Effective in predicting novel drug–target interactions
Machine learning	Protein–protein interaction networks	Accuracy: 93.8%	High accuracy in predicting drug–protein associations

Table 1 shows the related work of machine learning applications in drug–gene interactions.

**Table 1:** Top hub genes involved in the drug–gene interaction of type 4 collagen.

Sno	Protein ID	Gene
ENSP00000398698	P01375	TNF
ENSP00000269305	Q8J016	TP53
ENSP00000360266	P05412	JUN
ENSP00000381185	P10415	BCL2
ENSP00000405330	P03372	ESR1
ENSP00000226574	P19838	NFKB1
ENSP00000437955	Q16665	HIF1A
ENSP00000275493	Q9H2C9	EGFR
ENSP00000451828	P31749	AKT1
ENSP00000354558	P42345	MTOR

### 2.3. Cytoscape Networks

Using Cytoscape, datasets were imported, and a network graph was constructed for drugs and genes. The graph is then examined for structure, connectivity patterns, clustering coefficients, and centrality measures. The Cytohubba plugin can identify crucial hub genes within the network using maximum centrality clique methods. Data normalization and handling of missing values were conducted to mitigate the impact of larger variables.

### 2.4. Dataset Preparation

The dataset of drugs and genes was split into a training set (80%) for predictive modeling and a test set (20%) for performance evaluation. Machine learning algorithms, including neural networks and Naïve Bayes, were employed to analyze drug–gene interactions, facilitating prediction or classification tasks. Integrating these approaches with Python machine learning algorithms can provide a comprehensive understanding of drug–gene interactions and potentially reveal novel insights into type 4 collagen.

Neural networks and Naive Bayes are widely used for predicting drug–gene interactions [18, 19]. Neural networks excel at handling complex, nonlinear relationships within large datasets, enabling them to process large and high‐dimensional data effectively. They offer flexibility and adaptability, leading to accurate predictions. On the other hand, Naive Bayes, a probabilistic machine learning algorithm, efficiently manages high‐dimensional data and provides interpretable results. The selection between these two methods depends on the dataset’s characteristics and the desired trade‐off between accuracy and interpretability.

Neural networks and Naïve Bayes classifiers were chosen to represent two distinct machine‐learning paradigms for biomedical interaction prediction. Neural Networks model complex, nonlinear relationships among high‐dimensional features, capturing drug–gene interactions and pathways in Type IV collagen–mediated gingival epithelialization. Naïve Bayes served as a probabilistic baseline due to its efficiency, robustness with noisy data, and interpretability, enabling transparent assessment of feature–outcome links. Comparing these models allows evaluation of the trade‐off between predictive performance and interpretability, aiding methodological benchmarking in oral drug–gene interaction studies.

### 2.5. Neural Network Architecture

Machine learning algorithms, specifically neural networks and Naïve Bayes, were implemented using Python. The neural network architecture, comprising 10 hidden layers, ReLU activation function, Adam optimizer, regularization, and a maximum of 100 iterations, is a robust model for complex problem‐solving. This architecture includes an input layer, 10 hidden layers, and an output layer. The ReLU activation function introduces nonlinearity, enabling the model to capture complex patterns. The Adam optimizer adjusts the learning rate based on the gradient of the loss function, enhancing convergence speed. Regularization prevents overfitting by adding a penalty term to the loss function.

### 2.6. Naïve Bayes Architecture

A Naïve Bayes classifier is a straightforward architecture comprising a single layer with multiple nodes representing input data features or attributes. It applies Bayes’ theorem to calculate the probability of each potential class label, learns the probability distribution from training data, and predicts the class label with the highest probability. This simple architecture is efficient for text classification and email spam filtering. However, it may not perform optimally when dealing with highly dependent features or data that violate the independence assumption.

### 2.7. Evaluation Metrics

Machine learning models, such as Naive Bayes and neural networks, are evaluated using specific metrics to measure their effectiveness. These metrics include accuracy, precision, recall, F1 score, area under the curve–receiver operating characteristic (ROC) curve (AUC–ROC), and confusion matrix. Accuracy is the ratio of correctly predicted instances to the total instances, while precision is the ratio of correctly predicted positive observations to the total predicted positives. Recall is the ratio of correctly predicted positive observations to the total number of actual positives. The F1 score is the harmonic mean of precision and recall, balancing the two. The AUC–ROC measures the model’s ability to distinguish between classes at different threshold settings. The confusion matrix is valuable for evaluating classification algorithms and providing insights into TP, TN, FP, and FN. These metrics are typically calculated based on the predicted classes versus the actual labels from the dataset.

## 3. Results

### 3.1. Network Results

The dataset comprises 1917 drugs, each showing significant connections with various protein interactions and genes, resulting in a network with 372 nodes and 5180 edges. On average, each node is connected to ~28.152 neighbors. The network has a width of 5, meaning there can be at most five hops between any two nodes. Its radius is three, indicating that it takes a maximum of three hops to travel from the most central node to any other node. The characteristic path length of 2.381 suggests that, on average, it takes 2.381 steps to travel between any two nodes.

Nodes in the network tend to form clusters or communities due to their high interconnectivity, as indicated by the clustering coefficient of 0.490. The network density is 0.077, suggesting a moderately sparse network with only 7.7% of potential connections. The network’s heterogeneity value of 1.036 indicates significant heterogeneity in the node degree distribution.

**Figure 1:**
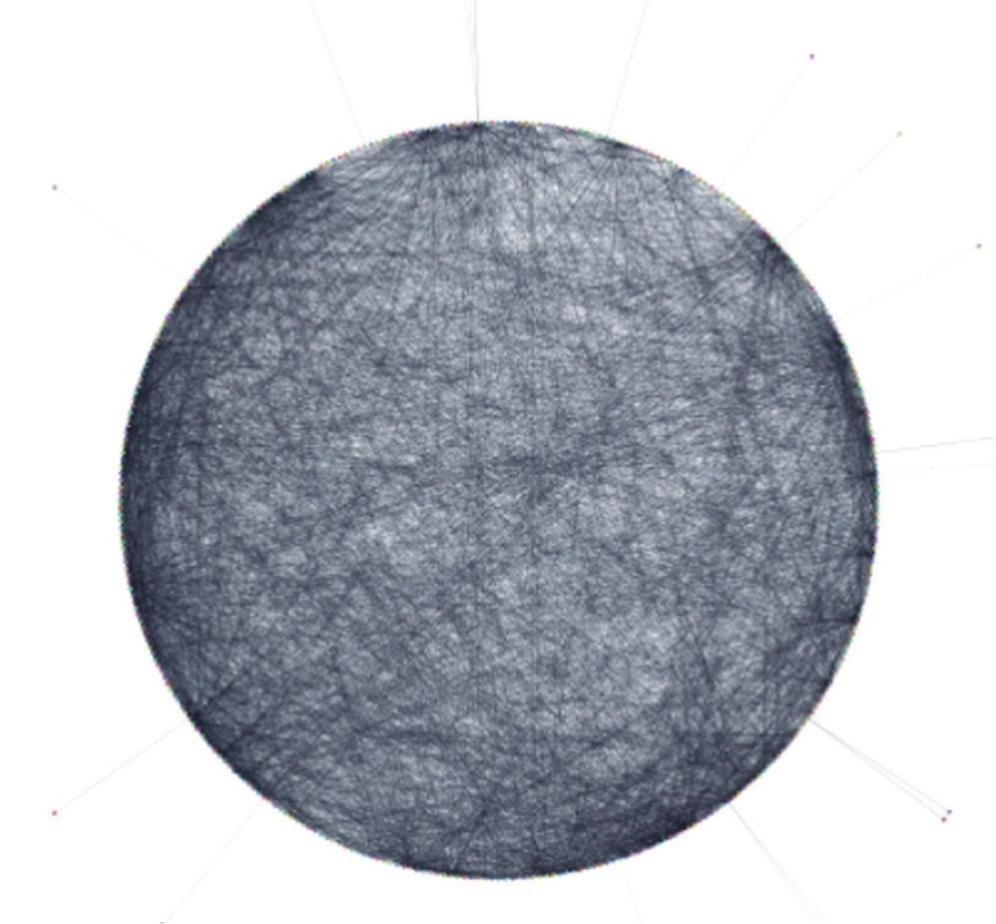
Network interactome of genes involved in type 4 collagen.

**Figure 2:**
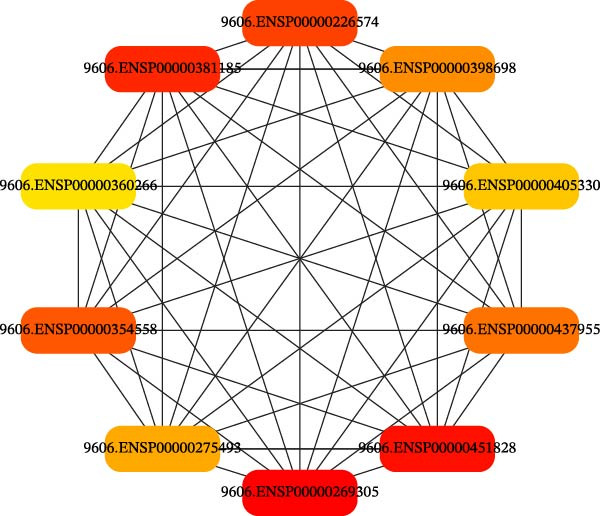
Identifying the top 10 hub genes using the cytohubba plugin with the maximum clique method.

The network’s centralization value is relatively low at 0.386, with no dominant central node. The network comprises five connected components, suggesting separate subgroups or separated areas. The network analysis was completed in ~0.238 s (Figures 1 and 2).

The results of predicting drug–gene interactions in type 4 collagen using Naive Bayes and Neural Network models are presented in Table 1.

Table 1 presents the top 10 hub genes in type 4 collagen‐drug gene interactions.

The Naive Bayes and neural network models exhibit high AUC values, indicating effective discrimination between positive and negative drug–gene interactions in type 4 collagen. However, the neural network model outperforms the Naive Bayes model regarding overall accuracy, F1 score, precision, and specificity. The Naive Bayes model has lower overall accuracy, while the neural network model has higher accuracy. The F1 score, which measures accuracy, is higher for both models, indicating better overall performance. Both models demonstrate high recall, but the Neural Network model performs better in terms of specificity. Further evaluation and interpretation are needed to inform decisions on drug–gene interactions involving type 4 collagen (Table 2).

**Table 2:** Performance metrics for Naive Bayes and neural network models.

Model	AUC	CA	F1	Precision	Recall	Specificity
Naive Bayes	0.995	0.109	0.197	0.969	0.109	1
Neural Network	0.975	0.969	0.953	0.938	0.969	0.031

**Figure 3:**
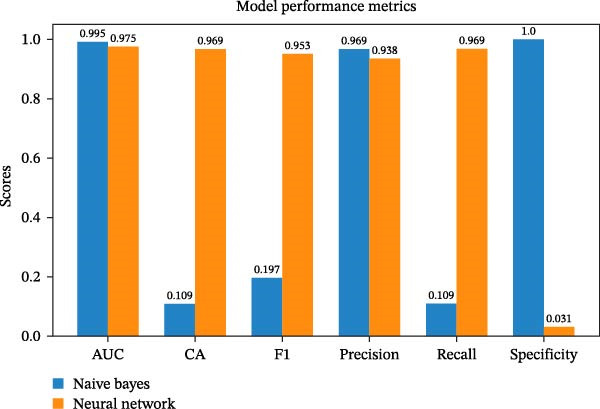
Model performance of the naive bayes and neural networks.

Figure 3 shows the model performance of the naïve bayes and neural networks.

Naive Bayes achieves a high AUC of 0.995, indicating excellent classification accuracy (CA). However, its CA is low at 0.109, suggesting that overall accuracy is also low. The F1 score, which balances precision and recall, is 0.197 for Naive Bayes, indicating a poor balance between the two. Despite having a high precision of 0.969, its low recall of 0.109 suggests it struggles to identify many positive samples. However, its specificity is 1, indicating proficiency in identifying negative samples.

The Wilcoxon signed‐rank test statistic was used for better interpretation, and the value was *p*‐value: 0.84375. The performance metric differences are not statistically significant, with a test statistic of 9.0 and a *p*‐value of 0.84375, which is significantly higher than the typical significance level of 0.05.

The performance metrics of the two models are not statistically significant, indicating that, despite individual differences, neither consistently outperforms the other across all metrics. This could be due to the small number of metrics compared and the mixed performance of the models across different metrics. Therefore, it’s advisable to be cautious about determining one model’s superiority solely on these metrics.

**Figure 4:**
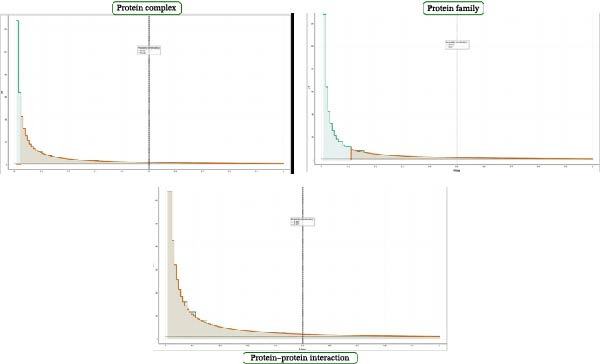
Lift curve of all classes. This figure shows three plots representing lift curves. The left plot shows a rapid decline in frequency, suggesting a concentration of data points at lower values. The middle plot shows a steeper decline, suggesting a more pronounced concentration of data at lower values. The right plot shows a more gradual decline, indicating a wider spread of data points. All three plots show a similar trend of decreasing frequency with increasing values, but differ in the steepness of the decline and in the spread of the data.

Naive Bayes outperforms the Neural Network model in terms of accuracy, precision, F1 score, and recall, but it has low specificity, which may lead to a high false‐positive rate (Figure 4).

**Figure 5:**
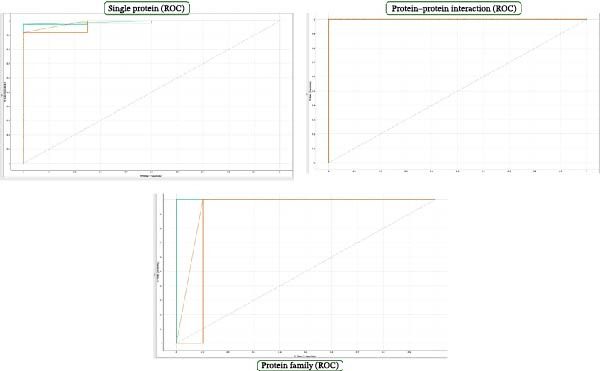
ROC curve of all classes.

Figure 5 shows three plots representing ROC or precision‐recall curves for different classification models or scenarios. The left plot shows a flat curve with a slight increase, suggesting poor performance. The middle plot shows a steep curve with no gradual increase, suggesting overfitting or poor performance on a specific subset of data. The right plot shows a steep rise followed by a more gradual decline, suggesting a better balance between sensitivity and specificity. The plots suggest varying levels of model performance, with the left plot indicating potential issues with classification. At the same time, the middle and right plots show better performance but may also indicate overfitting. Comparing the shapes and areas under the curves is crucial for evaluating classification models in machine learning.

**Figure 6:**
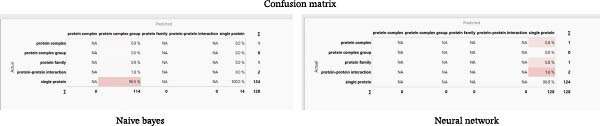
Confusion matrix of the naive Bayes and neural network models across all classes.

Figure 6 shows confusion matrices comparing the performance of Naïve Bayes and a neural network in categorizing protein types. Naïve Bayes correctly predicted single proteins 124 times (96.5% accuracy), while the neural network predicted complex groups and families once (0.9%). Both models showed high accuracy for single proteins but struggled with complex groups and families. The Naïve Bayes model had slightly higher accuracy for complex and PPIs than the neural network. Both models excel in identifying single proteins but struggle to distinguish other protein types.

Naïve Bayes has high AUC and precision but low accuracy and F1 due to class imbalance and threshold effects. High AUC doesn’t mean high accuracy across classes.

## 4. Discussion

Type 4 collagen is a crucial basement membrane component, providing structural support and acting as a physical barrier between the gingival epithelium and connective tissue [1, 20]. It forms a mesh‐like network that prevents detachment and maintains its position, while interacting with other components such as laminins and proteoglycans to support and regulate cell adhesion, migration, and tissue remodeling [21]. The hub genes identified in this study play a pivotal role in forming the gingival epithelial basement membrane.

Previous research has emphasized the importance of keratinized gingiva for oral health and hygiene, particularly in regulating the basement membrane keratinization process [1, 4]. It highlights the composition of type IV collagen. It suggests that the α1(IV):α2(IV):α5(IV) network, which includes the α6(IV) chain, is crucial for regulating keratinization in oral mucosal epithelial cells.

The hub genes identified play crucial roles in epithelization, including JUN, an AP‐1 complex transcription factor that regulates gene expression and cellular processes in the gingival epithelium [22]. BCL2, an anti‐apoptotic gene, maintains the epithelial barrier, prevents excessive cell death, and promotes tissue repair and wound healing [22]. ESR1 mediates estrogen effects in the gingival epithelium, regulating cell proliferation, differentiation, and inflammation. NFKB1 modulates the inflammatory response and immune function [23, 24], while HIF1A regulates cellular adaptations to hypoxia and wound healing [24, 25]. EGFR promotes cell growth and differentiation, while AKT1 regulates cell survival, proliferation, and tissue homeostasis. MTOR, a protein kinase, plays various roles in the gingival epithelium [26, 27]. It regulates cell growth, proliferation, and survival; maintains the integrity of the epithelial barrier; modulates inflammation; and promotes tissue repair and gingival wound healing.

High AUC values show both models effectively distinguish true drug–gene interactions from non‐interactions, aiding in prioritizing regulatory pathways in Type IV collagen–mediated epithelialization. The Naïve Bayes model’s high precision indicates strong confidence in predictions, reducing false positives and misleading hypotheses. Its low recall, however, means it might miss relevant but less common interactions. Conversely, the neural network has higher recall, detecting complex interactions better but with more false positives. This trade‐off shows a balance between discovering interactions and maintaining prediction reliability in drug–gene network modeling.

Drugs targeting these hub genes and their interactions are essential for novel drug design and to mitigate adverse metabolism. Previous studies have shown that drug–gene interactions can lead to genotype‐based predictions that affect drug metabolism, resulting in phenoconversion, where factors such as age, sex, and co‐medications may mismatch actual metabolism [28].

PPIs are crucial in cellular processes, and computational methods are required to predict them. PPIevo, an algorithm utilizing evolutionary feature extraction from protein sequences, has improved performance. However, it may overestimate performance due to negative dataset selection [29].

The accuracy of the Naive Bayes and neural network models is 99% and 97%, respectively, with class accuracies of 100% and 96% in predicting PPIs, single proteins, and protein families (Figures 1–6 and Tables 1 and 2). These predictions enable researchers to identify potential drug targets, understand drug mechanisms of action, and decipher complex biological pathways [30, 31]. Designed drugs disrupt specific protein interactions and assign functional annotations to uncharacterized proteins [32, 33].

Biologically, hub genes like TNF, EGFR, AKT1, MTOR, and NFKB1 serve as key regulators of inflammation, epithelial growth, and basement membrane changes in gingival tissue. Their strong predictive ability indicates drug–gene interactions affecting PI3K–AKT–mTOR and NF‐κB pathways are main drivers of Type IV collagen–mediated epithelialization. Clinically, targeting these pathways could improve gingival healing, reduce inflammation, and stabilize the epithelial barrier, underscoring the translational importance of computationally identified interactions. Future directions for improving the Naive Bayes model include incorporating advanced algorithms, exploring different neural network architectures, enhancing feature selection and engineering, and performing cross‐validation and hyperparameter tuning. However, limitations include an imbalanced dataset, generalizability, computational resources, interpretability, and a lack of feature information [34–36]. Techniques such as oversampling and undersampling, and evaluation metrics such as the area under the precision–recall curve, can address these limitations.

Communicative subgraph representation learning for multi‐relational inductive drug–gene interactions prediction (CoSMIG) is a novel method for predicting drug–gene interactions. It utilizes subgraph patterns without external information or retraining and outperforms existing models in transductive and inductive scenarios [37]. Additionally, assessing the model’s generalizability and computational resources can help improve its efficiency [35, 38, 39]. Furthermore, exploring explainable AI techniques or alternative interpretable models can improve the accuracy of identifying novel drugs that elicit better genetic responses in gingival type 4 collagen‐based epithelialization.

While no new machine‐learning architecture is proposed, this work evaluates model performance, parameter behavior, and network‐derived biological relevance in a domain with few predictive drug–gene interaction studies. The findings should be seen as methodological and hypothesis‐generating, serving as a foundation for future large‐scale, multi‐omics, or experimental research.

The study has limitations, including an imbalanced dataset, an assumption of feature independence, limited dataset size, reliance on predetermined features, lack of external validation, interpretability, model complexity, the model’s static nature, and a focus on type 4 collagen. The Naive Bayes algorithm may not capture complex biological interactions or dynamic biological changes over time. The study’s focus on type 4 collagen may limit its generalizability to other collagen types or to unrelated drug–gene interactions. Future work can address these limitations and build upon the findings, potentially leading to improved predictive models in drug–gene interaction research.

## 5. Conclusion and Future Directions

Predictions of drug–gene interactions for type 4 collagen offer significant potential for understanding biological pathways, identifying novel drug targets, designing targeted therapies, understanding disease mechanisms, and enabling personalized medicine approaches, thus promising effective treatments for gingival epithelization.

The Naive Bayes algorithm has been tested for predicting drug–gene interactions in type 4 collagen. Despite its high accuracy and precision, it may fail to identify positive samples and miss significant interactions, which are crucial for drug discovery and therapeutic implications. Future directions involve exploring advanced machine learning techniques to better accommodate biological data. Techniques such as ensemble methods, deep learning, oversampling/undersampling, domain‐specific knowledge, and semi‐supervised learning could enhance the model’s ability to identify important drug–gene interactions. Integrating multi‐omics data, including genomic, transcriptomic, and proteomic information, could provide a more comprehensive view of biological processes and interactions.

## Author Contributions

Shreya Arya, Deepavalli Arumuganainar, Pradeep Kumar Yadalam, and Carlos M. Ardila contributed to the conception, analysis, interpretation of data, and manuscript drafting. Shreya Arya, Deepavalli Arumuganainar, Pradeep Kumar Yadalam, and Carlos M. Ardila: Conceptualization. Pradeep Kumar Yadalam and Carlos M. Ardila: Methodology. Shreya Arya, Deepavalli Arumuganainar, Pradeep Kumar Yadalam, and Carlos M. Ardila: Data curation. Pradeep Kumar Yadalam, and Carlos M. Ardila: Writing‐ Original draft preparation. Shreya Arya, Deepavalli Arumuganainar, Pradeep Kumar Yadalam, and Carlos M. Ardila: Visualization. Pradeep Kumar Yadalam, and Carlos M. Ardila: Investigation. Shreya Arya, Deepavalli Arumuganainar, Pradeep Kumar Yadalam, and Carlos M. Ardila Validation. Pradeep Kumar Yadalam, and Carlos M. Ardila: Writing – Reviewing and Editing.

## Funding

No funding was received for this research.

## Disclosure

All authors agree to be accountable for the research presented.

## Conflicts of Interest

The authors declare no conflicts of interest.

## Data Availability

The data that support the findings of this study are available from the corresponding author upon reasonable request.
